# Phenotyping and metabolomics insights into the effect of melatonin in lettuce under non‐stress and salinity conditions

**DOI:** 10.1111/ppl.70055

**Published:** 2025-01-15

**Authors:** Elena Secomandi, Biancamaria Senizza, Marco Armando De Gregorio, Begona Miras‐Moreno, Rosa Maria Rivero, Pascual Garcia‐Perez, Luigi Lucini

**Affiliations:** ^1^ Department for Sustainable Food Process Università Cattolica del Sacro Cuore Piacenza Italy; ^2^ Department of Sciences, Technologies and Society Scuola Universitaria Superiore IUSS Pavia Pavia Italy; ^3^ Department of Plant Biology Faculty of Biology, University of Murcia, Campus de Espinardo Murcia Spain; ^4^ Department of Plant Nutrition Center of Edaphology and Applied Biology of Segura 12 (CEBAS‐CSIC) Murcia Spain; ^5^ Department of Food Technology, Nutrition and Food Science Veterinary Faculty, University of Murcia, Regional Campus of Internnational Excellence “Campus Mare Nostrum” Murcia Spain

## Abstract

Melatonin (MLT) is an indole derivative that exhibits hormone‐like activities in plants, regulating multiple aspects of growth and development. Due to its role in mitigating oxidative stress and facilitating osmoprotectant accumulation, MLT enhances abiotic stress tolerance, although the pathways and metabolic mechanisms involved remain unclear despite being studied in various crops. This work aimed to investigate the changes elicited by the exogenous MLT application at different concentrations (10, 50, 150 μM) and its role in mitigating the salinity stress in *Lactuca sativa* L. through metabolomics and phenotyping approaches. Our results clearly indicated that MLT increases photosynthetic efficiency at high dosage (150 μM) at either early or late salinity stress conditions (*p* < 0.01). Untargeted metabolomics provided insight into the significant effect of salinity and MLT (*p* < 0.01 in both cases, according to multivariate chemometrics), mediated by a broad reprogramming involving secondary metabolism, phytohormones, fatty acids and amino acids biosynthesis. In detail, 150 μM MLT induced an adjustment of the phytohormones profile to reduce the salinity‐induced damages. Our findings support the well‐known potential of melatonin in alleviating salinity stress. These findings address existing challenges in studying the molecular effects of MLT in mitigating abiotic stress, providing insights into the biochemical pathways that drive its effectiveness. In this sense, further research is acknowledged to provide a multidisciplinary high throughput perspective leading to its exploitation in a wide range of crops of agricultural and economic importance.

## 1 INTRODUCTION

Among environmental stressors, soil salinity is one of the most impactful abiotic stresses severely hampering agricultural production. The estimated area of salt‐affected soils is globally around ~8.31–11.73 Mkm^2^ due to both natural and anthropogenic causes, mainly related to improper agricultural practices (Hassani et al. 2021). The negative impact of salt stress depends on several factors, including the NaCl concentration, plant species, varieties and growth stages, and environmental conditions (Aghighi Shahverdi et al. 2018). In general, under salt stress, the inhibition of photosynthetic electron transport leads to the accumulation of reactive oxygen species (ROS), causing cell damage and oxidative stress. Also, higher levels of Na^+^ prompt the perturbation of Na^+^/K^+^ homeostasis in the cytoplasm with consequent inhibition of the correct cell functions, destabilization of cell membranes, and alteration of enzymatic activities (Kamran et al. 2020). In addition, the accumulation of sodium ions inhibits protein synthesis, leads to nutrient imbalance, and hampers the soil's organic potential and water uptake. The overall imbalance can thus damage the photosynthetic apparatus, impairing plants' photosynthetic performance (Mehta et al. 2010). The response to salt stress includes biochemical and molecular mechanisms at cellular and whole‐plant levels (Chen et al. 2018). At the cellular level, the adaptation comprises the selective absorption and exclusion of ions, the portioning of ions into the principal vacuole, the synthesis and increase of organic solutes in the cytoplasm, and changes in the composition of the membrane (Chen et al. 2018). At the plant level, the adaptation includes the regulation of the root's ion uptake, controlling ions transfer from roots to shoots and disseminating the ions from the shoots to different organs. Consequently, the photosynthetic pathway changes, the activity of antioxidant enzymes is modified, and the levels of plant hormones are altered (Liu et al. 2018).

Lettuce (*Lactuca sativa* L.) is an annual leafy crop that mainly grows in the world's temperate and sub‐tropic regions with a global production of more than 2 billion tons in 2022 (FAOSTAT), confirming its importance in the horticulture industry. Compared to staple crops, horticultural species are more susceptible to salinity stress and lettuce is considered a sensitive or moderately salt‐sensitive crop (Franzoni et al. 2022), depending on the cultivars (Xu and Mou 2015). The effect of salt stress has been widely documented and includes biomass reduction due to ionic imbalance and osmotic and toxic effects (Yildirim et al. 2015). Novel strategies should be undertaken to prevent those deleterious effects attributed to salinity on this crop.

Among the wide range of biostimulants established, melatonin (MLT, N‐acetyl‐5‐methoxytryptamine) has emerged as a promising agent to cope with the impact of abiotic stress in crops. MLT is a plant growth regulator which fulfils multiple physiological functions, including seed germination, growth, rooting induction, regulation of circadian rhythms, and mitigation of oxidative damage (Chen et al. 2009; Sarrou et al. 2014; Zhang et al. 2017). MLT is effectively involved in plant tolerance to abiotic stress by directly scavenging the free radicals, enhancing ROS detoxification, and regulating the enzymatic and non‐enzymatic antioxidant systems (Zhang et al. 2020). Moreover, it affects intracellular Ca^2+^ and the permeability of membranes (Li et al. 2016), and modifies the accumulation of osmoprotectants. Also, MLT regulates the genes involved in photosynthesis, carbohydrate and fatty acid biosynthesis, and nitrogen metabolism (Shi et al. 2015; Wei et al. 2018). It was proposed that treatments with MLT postponed leaf senescence by inhibiting senescence‐related genes (Liang et al. 2015).

Given the positive role of endogenous MLT in responses to stress and the ability to enhance the tolerance and survival of different plant species (Arnao and Hernández‐Ruiz 2015; Zhang et al. 2015), some studies have focused on the possible benefit of its application under salinity conditions (Li et al. 2017; Yan et al. 2021). By increasing endogenous abscisic acid (ABA) and gibberellic acid (GA) levels, exogenous MLT regulates the antioxidant system, hence inducing a higher salinity resistance (Zhang et al. 2014). In this regard, deciphering the role of MLT as a biostimulant agent to cope with abiotic stress, such as salinity, deserves further research facing its large‐scale application, becoming a convenient solution for the establishment of sustainable, more innovative crop systems with induced resilience in the current context of climate change crisis.

Despite being already studied in various crops (Huangfu et al. 2021; Ren et al. 2022; Yang et al. 2024), the pathways and intricated metabolic mechanisms underlying MLT‐mediated stress tolerance are fragmented while investigations of its role in plant adaptation and survival are gaining interest. As such, further research is necessary to delve into the complexities of these processes, deciphering the molecular and physiological processes mediated by MLT in the salt stress response. The application of an untargeted metabolomics approach is known to be highly useful in describing the effects at a phenotypical level, providing a detailed and broad perspective about the biochemical processes associated with plant stress mitigation and the effect of biostimulants in counteracting plant stress (Bernardo et al. 2019). In this work, *Lactuca sativa* L. var. canasta was grown under control and NaCl conditions and exposed to foliar‐spayed melatonin applications at three increasing concentrations (10, 50, 150 μM) to elucidate the mechanism of action of melatonin in the mitigation of salinity stress at a phenotypical level.

## 2 MATERIALS AND METHODS

### 2.1 Plant Material and Growth Conditions

Lettuce (*Lactuca sativa* L., cv. canasta) was grown in June 2022 at the facilities of Università Cattolica del Sacro Cuore (Piacenza, Italy). Seeds were soaked in distilled water for 1 h30 and placed on filter paper moistened with distilled water for 24 hours at room temperature in dark conditions for germination. Seedlings were then transplanted into pots (6 x 6 x 9.5 cm) filled with 130 g of a peat substrate (Radicom, Vigor Plant), and placed in a growth chamber at 20°C ± 2°C, 16/8 h light/dark photoperiod, and at photosynthetic photon flux density (PPFD) of 360 μmol m^−2^ s^−1^ (Ambralight) (Sublett et al. 2018). Lettuce seedlings were watered daily with 30 mL of water per pot for 14 days until the third‐leaf growth stage.

**FIGURE 1:**
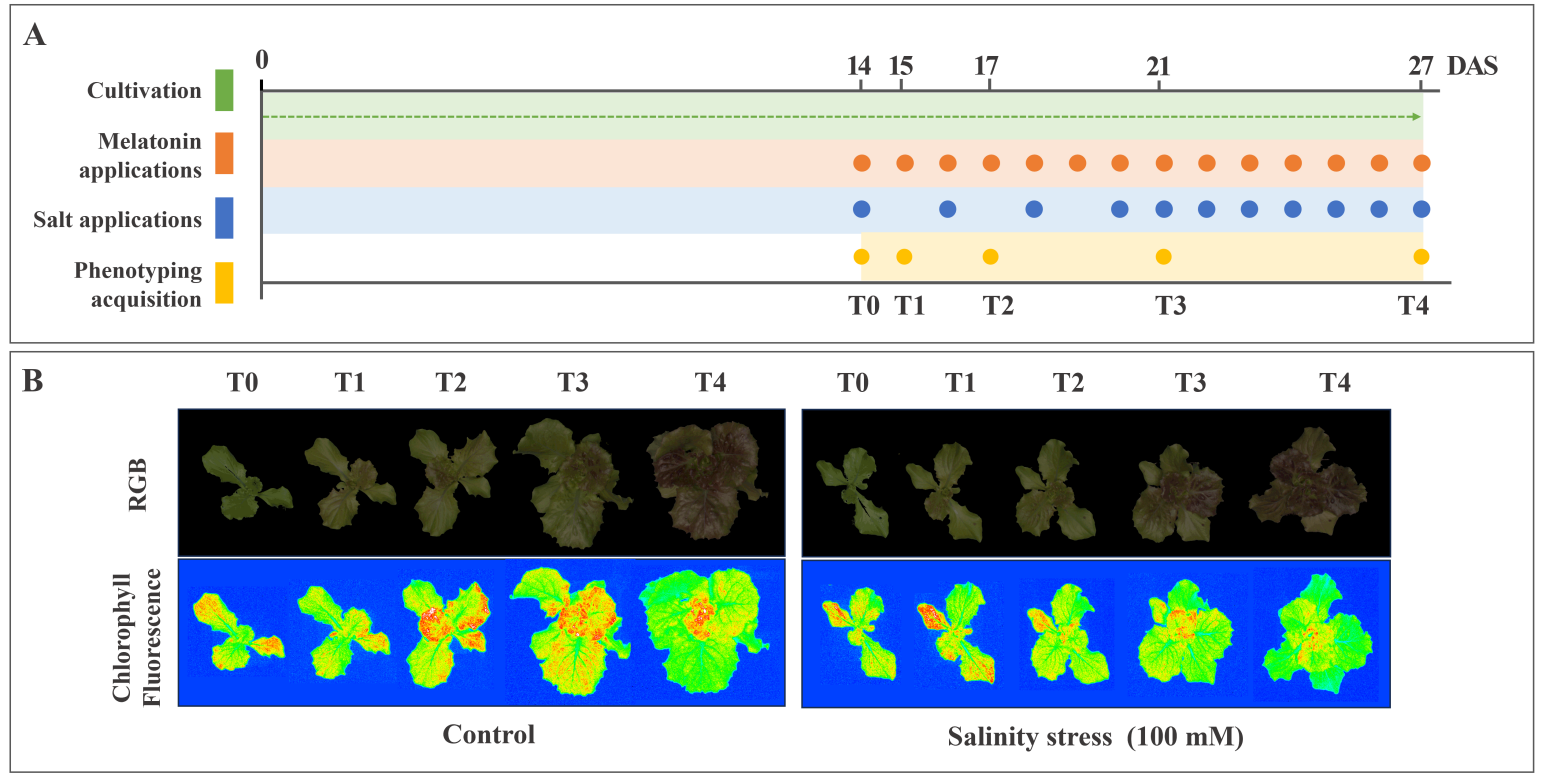
**A**. Experimental design of Lactuca sativa L. cv Canasta samples treated with different MLT concentrations under salinity and non‐salinity conditions. The dots correspond to the days of application of MLT and NaCl and the data acquisition with the PlantScreen System. **B**. RGB images and ChlF images for control and salinity‐stressed lettuce at T0, T1, T2, T3, and T4 corresponding to 14th DAS (before the beginning of the treatments and at 15^th^, 17^th^, 21^st^, and 27^th^ DAS). Upper: color‐segmented top view Red Green Blue (RGB) images; Lower: false‐color images of maximum fluorescence values in a dark‐adapted state (Fv/Fm).

The experimental design consisted of eight treatments with ten biological replicates per treatment. Specifically, on the 14th day after sowing (DAS), plants were randomly distributed into two groups, corresponding to salinity‐stressed and non‐stressed conditions (Figure 1). The stress was applied by watering plants with 30 mL of 100 mM NaCl solution on alternate days for 7 days and then daily for 7 additional days. Control plants were watered with 30 mL of distilled water, with the same schedule used for salt‐stressed plants. Melatonin (Sigma‐Aldrich) was foliar‐sprayed daily on salinity‐stressed and non‐stressed plants from the 14th DAS until the end of the experiment at concentrations of 10, 50 and 150 μM. Control plants were sprayed with water.

### 2.2 RGB imaging and Chlorophyll Fluorescence measurement

During the 14 days of treatment, digital biomass and photosynthetic performance were detected through the PlantScreen™ System (Photon System Instruments). The analysis was conducted at different growing stages to monitor lettuce growth rates and chlorophyll fluorescence. Specifically, the measurements started at 14‐day‐old plants and were recorded at 14, 15, 17, 21 and 27 DAS, corresponding to T0, T1, T2, T3, and T4 (Figure 1A‐B). Plant image acquisition was performed based on the Fv/Fm protocol by PlantScreen™. Briefly, the plant masks were manually drawn to acquire the entire leaf area surface according to what was captured by the RGB2 top view camera in the plant screen system (GigE PSI RGB, 12.3 Megapixels with 1 / 2.3” CMOS SENSOR) and, before the ChlF images acquisition, the plants underwent a period of 15 minutes dark‐adaptation. Then, the MorphoAnalyser software (version 1.0.9.6) was used to examine the collected images and assess the total biomass area of the lettuces. Specifically, the total number of pixels acquired was successively converted to mm^2^. Correct. The plant mask were manually drawn to acquire the entire leaf area surface according to what captured by the RGB2 top view camera in the plant screen system.

Chlorophyll fluorescence imaging of induction kinetics Chlorophyll (Chl) fluorescence was measured using the FluorCam 7 application imaging system (Photon Systems Instruments). Chl fluorescence images and induction kinetics were measured on pre‐darkened leaves (15 min) using the FluorCam Fv/Fm protocol. The measured Chl fluorescence intensity images were obtained in false colours, where black is the lowest (zero) and red is the highest fluorescence intensity. The fluorescence emission is induced by two sets of 325 super‐bright orange light emitting diodes (LEDs) (620 nm) that provide excitation flashes or a continuous actinic irradiance controlled by a defined protocol. Fluorescence images are captured by a CCD camera at 16‐bit resolution in 1360 x 1024 pixels of CCD chip. Chlorophyll fluorescence images of parameters F0 and F0’ (minimum fluorescence in the dark and the light‐adapted states), Fm and Fm′ (maximum fluorescence in the dark and the light‐adapted states) were recorded during induction kinetics. Images of various Chl fluorescence ratios obtained by pixel‐to‐pixel arithmetic operations performed by FluorCam software were converted into Photosystem II maximum quantum yields Fv/Fm.

### 2.3 Growth measurements: Fresh weight (FW), dry weight (DW), Dry matter percentage (DM%)

At the end of the experiment, five plant replicates of leaves and roots were used to assess the biomass, while the remaining five were deep‐frozen in liquid nitrogen to stop the metabolism and stored at −20°C for subsequent metabolomics analysis. Fresh weight (FW) was achieved by weighting leaves and roots with a digital precision balance. Samples were successively dried at 60°C, and dry weight (DW) was measured. Leaf dry matter percentage (DM%) was calculated as DM = (shoot dry weight/shoot fresh weight) × 100.

### 2.4 UHPLC‐ESI/QTOF untargeted metabolomics

Each sample was obtained by weighing 1 g of powdered leaves. Metabolites were mechanically extracted using a Politron homogenizer (IKA T10) for 2 minutes at room temperature. The extractive solution was composed of 80% methanol (MeOH, purity ≥99.8%, Sigma‐Aldrich) with 0.1% (v/v) formic acid (purity ≥95%, Sigma‐Aldrich). The samples were centrifuged at 10′000 *g* for 15 minutes at 4°C (Eppendorf 5810R) and 1 mL of the resulting supernatant was filtered in a vial with 0.22 μm regenerated cellulose filters. 5 replicates were prepared for each condition.

The secondary metabolites were determined through ultra‐high‐pressure liquid chromatography (UHPLC) coupled to electrospray quadrupole‐time‐of‐flight hybrid mass spectrometry (UHPLC‐ESI/QTOF‐MS), as previously described by Senizza et al., (2023). Measurements were performed using a 1290 liquid chromatography system equipped with a binary pump and a Dual Electrospray JetStream ionization source coupled to a G6550 mass spectrometer detector (all from Agilent Technologies). The mass spectrometer operated in Full‐Scan mode with positive ionization (ESI +) and accurate masses in the 100–1200 m/z range. The chromatographic separation was performed through an Agilent Zorbax Eclipse plus C18 analytical column (15 cm x 2.1 mm, 1.7 μm particle size), using water‐acetonitrile gradient elution (from 6% up to 94% in 34 min), with an injection volume of 6 μL, no analytical replications.

Compounds annotation was reached through the database PlantCyc by combining the monoisotopic accurate mass and isotopic pattern (i.e., isotope spacing and ratio) and considering a mass accuracy <5 ppm (Hawkins et al. 2021). The software used was Profinder B.07 (Agilent Technologies), and the reference database was exported from PlantCyc 9.6. Level 2 of identification (i.e., putatively annotated compounds, COSMOS standards in metabolomics) was achieved (Salek et al. 2013). Only those compounds identified within 100% of replications in at least one treatment were retained and used for further statistics and chemometrics. The comprehensive instrumental conditions are presented in supplementary material.

### 2.5 Statistical analysis

Morpho‐physiological and photosynthetic data were analysed with the SPSS 28 software (IBM) for ANOVA (*p* = 0.05). Values are presented as mean ± standard deviation (*n* = 5). Metabolomics data were analysed through Agilent Mass Profiler Professional B.12.06 software, as described in previous works (Senizza et al. 2023). Compounds were log2 transformed, normalized at the 75th percentile and baselined against the median of each compound in the dataset. Then, the unsupervised hierarchical cluster analysis (Euclidean distance, Ward's linkage) was performed to naively investigate patterns across treatments. After that, the dataset was exported into the software SIMCA 13 (Umetrics) and elaborated for orthogonal partial least squares discriminant analysis (OPLS‐DA) supervised modelling. The model was cross‐validated using Cross‐Validation ANOVA, and the permutation testing was done to exclude overfitting (200 random permutations). Goodness‐of‐fit (R^2^Y) and goodness‐of‐prediction parameters (Q^2^Y) were recorded to evaluate the overall quality of the prediction models. Thereafter, the VIP (variables importance in projection) selection method was applied to select those metabolites possessing the highest discrimination potential (VIP score >1.2). Finally, the differential compounds obtained from the ANOVA and fold‐change analysis (*p* < 0.05, Bonferroni multiple testing correction and Fold‐Change ≥2) were exported into the Omic Viewer Pathway Tool of PlantCyc (Plant Metabolic Network) software for interpretation (Karp et al. 2009).

The supervised multivariate analysis based on analysis of variance (ANOVA) multi‐block orthogonal partial least squares (AMOPLS) was achieved using the “rAMOPLS” package (version 0.2) in R (version 4.2.3), as previously described (Boccard and Rudaz 2018). The log2‐transformed abundances of annotated features were subjected to AMOPLS modelling to determine the ANOVA‐mediated discriminant power of each factor. In detail, “MLT” for treatment, “Stress” for the stress group and “MLT x Stress” for their interactions were considered. Models were constructed by executing 100 permutations. The results for modelling were obtained after the selection of the optimal number of orthogonal components calculated, according to the model exhibiting statistical significance (α = 0.01) with the smallest number of orthogonal components. The model interpretation was based on the following parameters: (i) the residual structure ratio (RSR) that corresponds to the ANOVA consistency of each effect with respect to residuals, assuming that the highest values correlate with the highest contribution of each effect; (ii) p‐value; (iii) the principal predictive components (in percentage) representing the major block contributions associated with each effect; and (iv) the orthogonal component (in percentage) explaining the contribution of the orthogonal predictive component. AMOPLS models were also considered for the variable importance in the projection (VIP), presented by VIP^2^ markers, corresponding to those metabolites showing the strongest discrimination power of each effect involved in the model. For each significant effect, the top 50 VIP^2^ markers (exhibiting the highest VIP score) and their logFC values were used to allow pairwise comparisons.

## 3 RESULTS

### 3.1 Plant biomass and leaf digital area

Biomass of leaves and roots was measured after 14 days of MLT treatment and stress application (corresponding to 27th DAS) (Table S1; Table 1). As expected, salinity was the main factor impacting plant growth. An overall decrease (~20%) of fresh leaf biomass was observed in salt‐stressed plants compared to the non‐stressed ones, while no significant differences between control and NaCl‐treated plants could be outlined for dry leaf biomass (Table S1). Salinity stress also impacted root biomass with a reduction of ~31% of the FW and ~ 28% of DW, respectively. Considering MLT treatments, no dose‐dependent effects on biomass were observed (Table S1), while the simultaneous NaCl and MLT application strongly affected roots FW and DW of lettuces (Table 1). Surprisingly, roots treated only with 10 μM displayed the highest FW value in non‐stress conditions, but the lowest values were recorded under NaCl conditions. Finally, the combined treatments had non‐significant results on leaves FW and DW. DM% was statistically significant in salt and unstressed plants, with an increase of ~23% in stressed lettuce samples (Table S1). Considering MLT and salinity treatments, higher values were reported by NaCl +10 MLT samples, while lower results were that of plants foliar‐sprayed only with 10 μM MLT, in the opposite way as reported for root FW.

**TABLE 1:** ANOVA on the effect of melatonin (10, 50, 150 μM) on lettuce growth under non‐salt and salinity (100 mM) conditions. The symbol: “ns” = non‐significant, asterisks (*, **, ***) = significant at p ≤ 0.05 0.01, and 0.001, respectively. Within a column, letters (a–c) indicate different statistical groups according to Duncan's multiple range test (*p* = 0.05).

Treatment	Leaf Fresh Weight	Leaf Dry Weight	Root Fresh Weight	Root Dry Weight	Leaf DM%
	g plant^−1^	g plant^−1^	g plant^−1^	g plant^−1^	%
Control	8.30 ± 1.86	0.67 ± 0.16	6.32 ± 1.16^a^	0.40 ± 0.13^a^	8.10 ± 0.51^bc^
10 MLT	8.22 ± 1.68	0.59 ± 0.10	6.84 ± 0.94^a^	0.33 ± 0.06^ab^	7.41 ± 1.41^c^
50 MLT	7.94 ± 2.14	0.67 ± 0.22	6.00 ± 0.25^a^	0.32 ± 0.06^ab^	8.36 ± 0.75^bc^
150 MLT	8.12 ± 1.69	0.68 ± 0.14	6.52 ± 0.77^a^	0.36 ± 0.77^ab^	8.43 ± 0.86^bc^
NaCl	6.52 ± 1.33	0.66 ± 0.13	4.82 ± 0.85^b^	0.29 ± 0.10^ab^	10.16 ± 0.84^a^
NaCl +10 MLT	7.18 ± 1.74	0.74 ± 0.19	4.16 ± 0.88^b^	0.26 ± 0.09^b^	10.26 ± 0.41^a^
NaCl +50 MLT	6.48 ± 1.99	0.62 ± 0.23	4.56 ± 0.70^b^	0.26 ± 0.03^b^	9.46 ± 1.63^ab^
NaCl +150 MLT	5.82 ± 2.00	0.58 ± 0.20	4.20 ± 0.86^b^	0.25 ± 0.07^b^	9.92 ± 0.78^a^
	n.s.	n.s.	***	*	***

**FIGURE 2:**
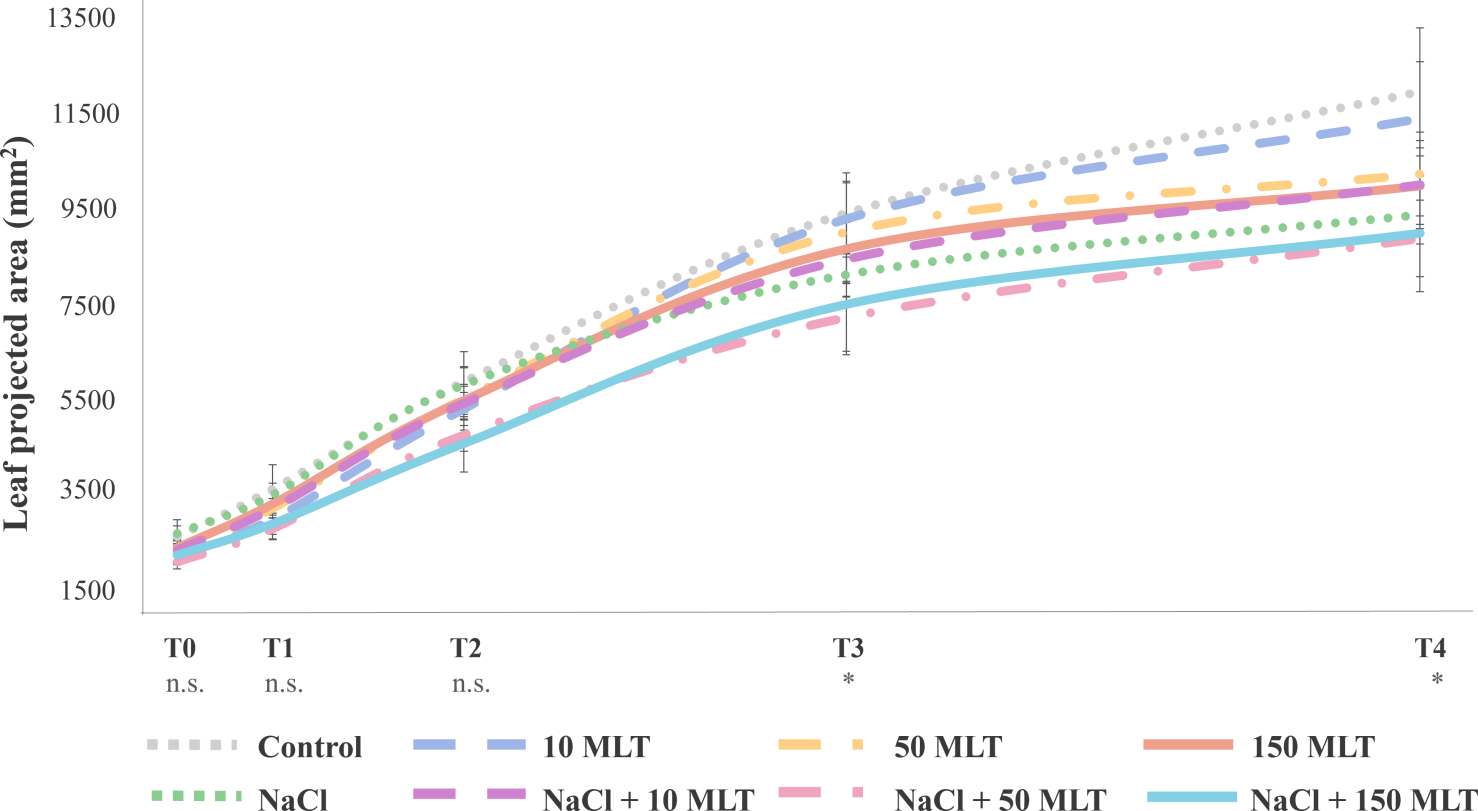
Leaf Projected area in different conditions at different time points (T0‐T4). Data were analyzed through one‐way ANOVA using Duncan's test as post hoc (*p* ≤ 0.05), *n* = 80. Each color corresponds to a distinct condition; no significant differences were observed during acquisition. The symbols: n.s. = non‐significant, * = significant at *p* ≤ 0.05.

To assess the effect of MLT on the lettuce growth status under both stressed and control conditions, the projected area (pixel) was acquired by the PlantScreen™ System (PSI) at different stages of the experiment, namely T0, T1, T2, T3, T4 (Figure 1), and then converted to mm^2^. Salinity affected the lettuce projected area starting from the first week of treatment. Indeed, the area of salt‐stressed plants was statistically different in T3 (with a decrease of 13.9%) and T4 (14.4% reduction) compared to control (Table S2). However, despite the progressive increase in the projected leaf area outlined in Figure 2, no significant effect could be observed concerning MLT treatment and the interaction between the MLT treatment and salinity stress (Table S2). Specifically, the maximum projected area was observed in control and 10 MLT plants; the NaCl +10 MLT lettuce showed comparable results with 50 MLT and 150 MLT plants, while the lowest values were recorded in NaCl +50 MLT and NaCl +150 MLT (Figure 2). These results are in line with the destructive plant biomass analysis carried out at the end of the experiment (Table 1). Indeed, leaves FW were strongly correlated (0.91, *p* = 4.288e^−16^ Pearson correlation) with the projected above‐ground area obtained through the RGB camera at T5 (corresponding to the harvesting day), with no deviation from a linear relationship (Figure S1).

### 3.2 Photosynthetic performance

To assess the effect of MLT application on the photosynthetic performance of lettuce, an automated chlorophyll fluorescence imaging setup was adopted (Table 2). Apart from T0, the photosynthetic performance of the plants was significantly affected starting from early stages (T1), with distinctive effects as a function of treatment and time (Table 2 and Figure S2). In fact, significant effects could be observed by separately investigating the effect of salt stress, melatonin application and their interaction. At T1 and T2, MLT application was more effective at the highest concentration, while it did not affect Fv/Fm in the second half of the test period (Table 2). Surprisingly, salt treatment had a slight but positive effect on QY at T2 and T3, which became insignificant at the end of the experiment (Table 2). Finally, considering the salinity x MLT interaction, the PSII maximum quantum yield showed distinct trends. Specifically, most of the combined treatments (e.g., 10 MLT, 150 MLT, NaCl) led to a significant reduction of the PSII maximum quantum yield during the experimental period, while NaCl +50 MLT led to progressive increase. Interestingly, lettuce foliar‐sprayed with 50 μM had lower values throughout almost all the testing period, whereas salt‐stressed plants under 150 μM MLT always showed a higher photosynthetic performance (Table 2).

**TABLE 2:** Effect of melatonin concentrations (10, 50, 150 μM) on lettuce under control and salinity (100 mM) conditions on the maximum quantum yield of photosystem II (Fv/Fm) at T0, T1, T2, T3, and T4. The symbol: “ns” = non‐significant, asterisks (*,**, ***) = significant at p ≤ 0.05, 0.01, and 0.001, respectively. Within a column, different letters (a–c) indicate different statistical groups according to Duncan's multiple range test (p = 0.05).

	T0	T1	T2	T3	T4
**Salinity (S; mM NaCl)**					
Control NaCl	0.791 ± 0.006	0.799 ± 0.006	0.808 ± 0.014	0.795 ± 0.008	0.812 ± 0.016
100 mM NaCl	0.790 ± 0.008	0.797 ± 0.009	0.822 ± 0.011	0.807 ± 0.006	0.812 ± 0.008
	n.s.	n.s.	***	***	n.s.
**Melatonin (MLT; μM)**					
0	0.792 ± 0.004	0.800 ± 0.005^a^	0.817 ± 0.018^ab^	0.798 ± 0.008	0.805 ± 0.008
10	0.788 ± 0.008	0.798 ± 0.006^a^	0.814 ± 0.014^ab^	0.802 ± 0.012	0.806 ± 0.006
50	0.790 ± 0.009	0.790 ± 0.006^b^	0.805 ± 0.009^b^	0.802 ± 0.006	0.811 ± 0.006
150	0.793 ± 0.005	0.804 ± 0.007^a^	0.826 ± 0.009^a^	0.801 ± 0.011	0.811 ± 0.010
	n.s.	***	*	n.s.	n.s.
**S ‐ MLT**					
Control	0.790 ± 0.005	0.800 ± 0.003^ab^	0.802 ± 0.009^bc^	0.795 ± 0.011^cd^	0.802 ± 0.005^c^
10 MLT	0.787 ± 0.009	0.800 ± 0.005^ab^	0.801 ± 0.006^bc^	0.792 ± 0.008^d^	0.806 ± 0.006^c^
50 MLT	0.795 ± 0.005	0.792 ± 0.004^bc^	0.799 ± 0.008^c^	0.799 ± 0.006c^d^	0.806 ± 0.004^c^
150 MLT	0.793 ± 0.004	0.803 ± 0.006^a^	0.827 ± 0.010^a^	0.793 ± 0.008^d^	0.803 ± 0.008^c^
NaCl	0.794 ± 0.003	0.800 ± 0.007^ab^	0.831 ± 0.008^a^	0.801 ± 0.004^bcd^	0.809 ± 0.010^bc^
NaCl +10 MLT	0.789 ± 0.009	0.796 ± 0.008^abc^	0.827 ± 0.005^a^	0.811 ± 0.006^a^	0.807 ± 0.006^c^
NaCl +50 MLT	0.785 ± 0.009	0.788 ± 0.007^c^	0.811 ± 0.007^b^	0.805 ± 0.003^abc^	0.816 ± 0.003^ab^
NaCl +150 MLT	0.794 ± 0.006	0.804 ± 0.009^a^	0.825 ± 0.010^a^	0.809 ± 0.007^ab^	0.818 ± 0.006^a^
	n.s.	**	***	***	**

### 3.3 Metabolomics analysis

Untargeted metabolomics was used to investigate the effect of the treatment with melatonin on the metabolomic profile of lettuce leaves in both stressed and control conditions. This approach allowed us to putatively annotate more than 2000 compounds used to explore the biological processes involved in plant responses. The annotated metabolite list, with composite mass spectra and abundance, is provided as supplementary material (Table S3), whereas the raw data are published in the repository MetaboLights (Yurekten et al. 2024) under study ID MTBLS10367.

**FIGURE 3:**
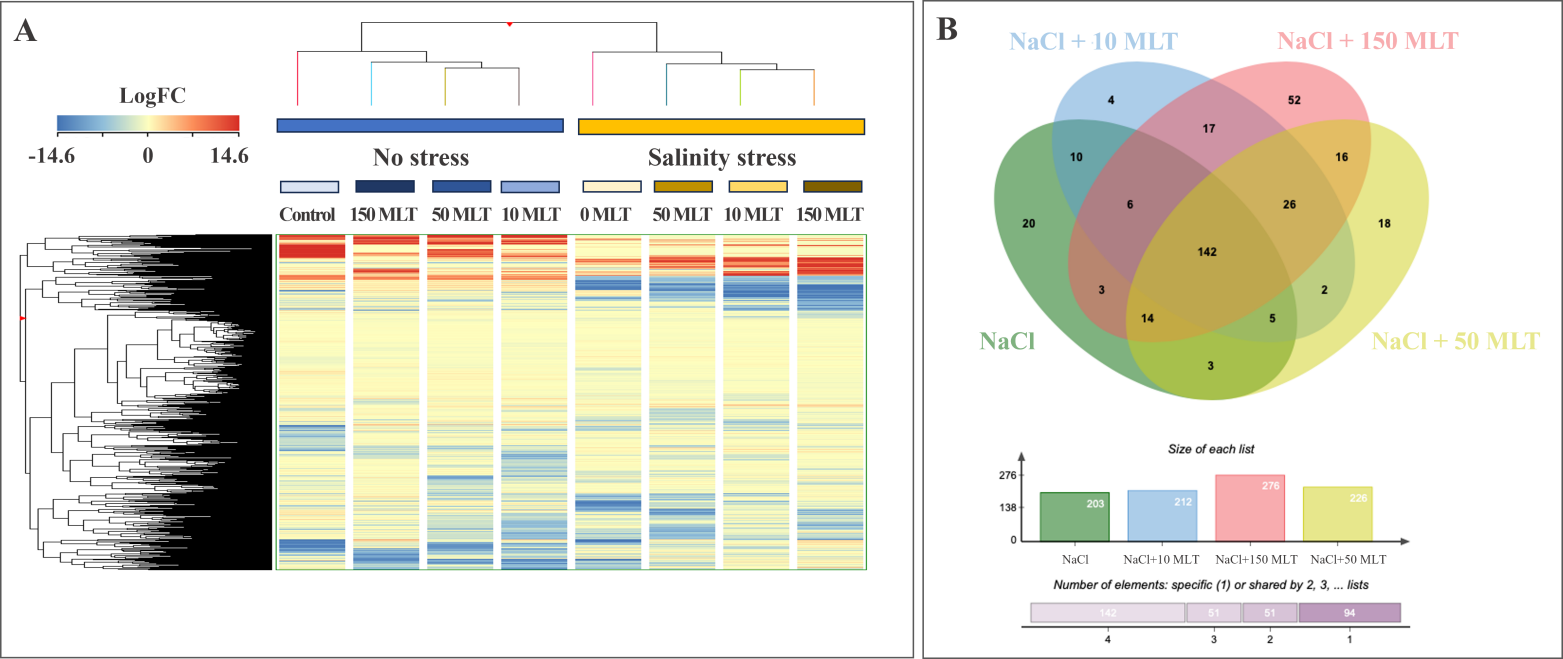
**A**. Unsupervised hierarchical cluster analysis of the metabolomic profiles of lettuce leaves, obtained by UHPLC/QTOF‐MS untargeted analysis, as a function of the salt stress and MLT treatments. Samples were clustered according to Ward's algorithm and based on Euclidean distances. **B**. The differential compounds from Volcano Plot analysis (*p* < 0.05; FC >2) of salinity‐stressed samples were considered to build the Venn diagram.

Firstly, the unsupervised hierarchical cluster analysis (HCA) provided a hierarchical picture of the different factors under study (Figure 3A), primarily highlighting a distinct metabolomic profile between lettuce growth in salinity and non‐stressed conditions. Considering the control samples, 10 and 50 MLT showed a similar phytochemical profile, different from the control (not treated with MLT), whereas plants under salinity pointed out a stronger effect of 150 MLT (Figure 3A). To better understand whether the salinity‐stressed samples treated with different concentrations of MLT had a shared metabolic response or differed in the production of compounds, a Venn analysis (Figure 3B) was carried out starting from the metabolites identified by Volcano Plot analysis (*p* < 0.05; FC >2). Among these, 142 metabolites were in common in all NaCl‐stressed samples, 52 were attributed only to the highest MLT concentration (NaCl +150 MLT), 18 were characteristic of the NaCl +50 MLT plants, and only 4 compounds were differently identified at the lower concentration (NaCl +10 MLT). On these bases, a dose–response effect could be observed at the metabolome level.

**FIGURE 4:**
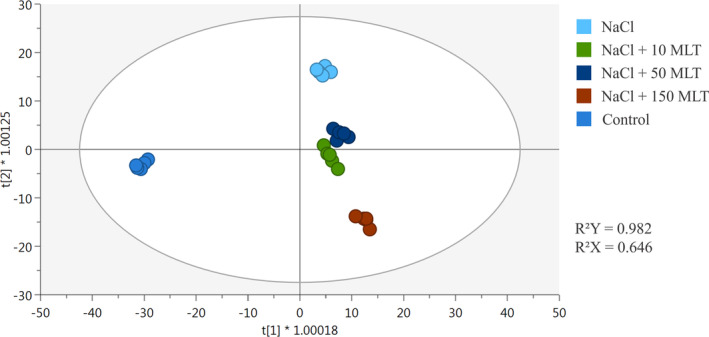
Score plot of orthogonal projection to latent structures discriminant analysis (OPLS‐DA) supervised modelling carried out on untargeted metabolomics profiles of lettuce in salinity conditions and treated with different concentrations of MLT (R2Y = 0.98, Q2Y = 0.64).

The supervised OPLS‐discriminant analysis, including control and MLT‐stressed samples, further confirmed these findings. The model score plot (R^2^Y = 0.982; Q^2^Y = 0.62) separated the salinity‐treated samples, underlining differences in the metabolomic signature between samples (Figure 4). Although the main separation was between control and stressed samples, confirming the HCA output (Figure 3A), variations due to different MLT doses could be recorded. Notably, 150 MLT induced the most significant change, while 10 or 50 MLT had minimal impact on the metabolome profile, consistent with the Venn analysis.

**FIGURE 5:**
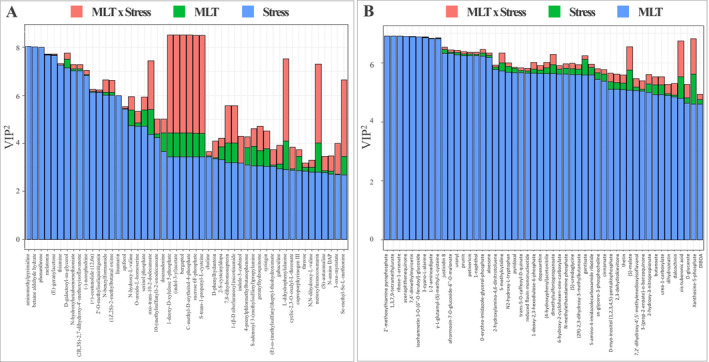
The Variable importance in projection (VIP2) analysis from AMOPLS. **A**. represents the metabolites mainly affected by the salinity stress; **B**. represents the metabolites affected by the MLT treatment. Discriminant compounds with a VIP score >1.2 are reported in Table S4.

Then, the VIP analysis was employed to evaluate the compounds driving this separation. The compounds considered, having a VIP score >1.2, were 145 (Table S3) and included oxidized fatty acids (traumatin), glycosylglycerol, amino acids (asparagine, L‐dihomomethionine), terpenoids ((E)‐geranylacetone, a‐cyclocostunolide, capsidiol) and polyphenols (apiforol, tyrosol, salvianin). AMOPLS was performed to unravel the metabolites influenced by the interaction between MLT treatment and salt stress, using MLT, NaCl and MLT x NaCl as model interpretations. Only the top‐50 VIP^2^ markers related to MLT and NaCl were retained to uncover the metabolites contributing to the differences between treatments and NaCl (Figure 5). In general terms, compounds belonging to flavonols, amino acids, nitrogen and sulfur compounds, mainly thiamine pyrophosphate, theacrine, 7,4′‐dimethylmyricetin and isorhamnetin 3‐*O*‐(6”‐*O*‐feruloyl)‐glucoside were the compounds with the strongest effect in the discrimination of stressed samples.

The discriminant VIP^2^ obtained following melatonin treatment were aminomethylpirimidine and betaine aldehyde hydrate, both primarily responsive to NaCl. Lastly, the analysis highlighted the compounds involved in MLT x NaCl interaction, pointing out the melatonin‐mediated response to stress. Among these latter, carbohydrate derivatives (2‐C‐methyl‐D‐erythritol 4‐phosphate, deoxyxylulose 5‐phosphate and sucrose 6‐phosphate), the auxin indole‐3‐acetate, the furanocoumarin bergaptol, as well as the cysteine derivatives Se‐methyl‐selenocysteine and propenyl‐L‐cysteine, could be identified as main discriminants.

**FIGURE 6:**
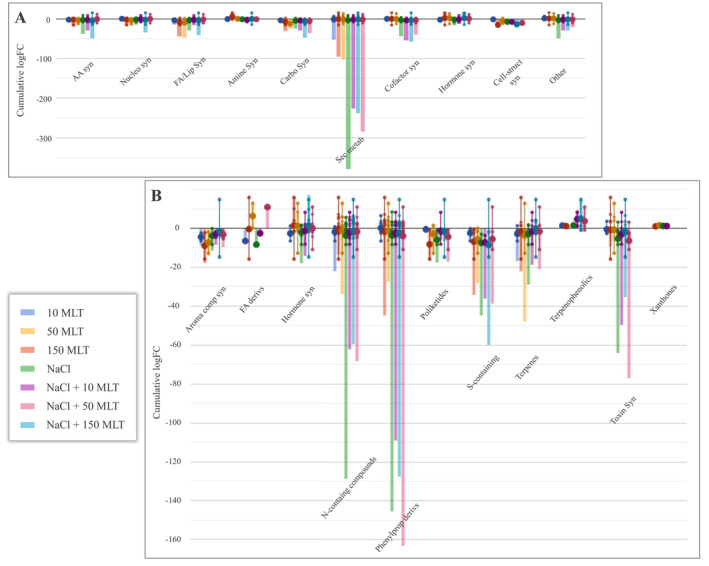
**A**. Metabolic pathway analysis and **B**. the relative details of secondary metabolism of lettuces treated with 100 mM NaCl and 10, 50, 150 μm MLT compared to control. The compounds selected by Volcano Plot analysis (Log FC = 2, p‐value ≤0.05, were uploaded into the PlantCyc pathway tool1. The x‐axis represents each set of metabolic subcategories, while the y‐axis corresponds to the cumulative log fold change (FC) of compounds within each category. The mean of all FCs for the different metabolites in the class is represented by the big dots, while the individual log FC is visualized via the small dots. AA syn, amino acid biosynthesis; Nucleo syn, nucleoside and nucleotide biosynthesis; FA/Lip syn, fatty acid and lipid biosynthesis; Amine Syn, amine biosynthesis; Sec metab, secondary metabolite biosynthesis; Cofactor syn, cofactor, carrier, and vitamin biosynthesis; Hormone syn, Hormones biosynthesis; Cell‐struct syn, cell structure biosynthesis; Aroma comp syn, Aromatic compound biosynthesis; FA deriv, fatty acid derivates; N‐containing, nitrogen‐containing secondary compound biosynthesis; Phenylprop derivs, phenylpropanoid derivative biosynthesis; S‐containing, sulfur‐containing secondary compound biosynthesis.

After that, the differential compounds of each treatment comparison from the Volcano Plot analysis (Log FC >2, *p* < 0.05) were used for the pathway analysis. The results (Figure 6) showed a robust down accumulation of metabolites related to secondary metabolisms and compounds involved in amino acids, fatty acids, and carbohydrate biosynthesis. In detail, nitrogen‐containing compounds were down‐accumulated in all the conditions under investigation, mainly under salinity. Still, MLT application triggered an accumulation of this class of compounds. Concerning phenylpropanoids, a specific modulation could be observed as a function of the treatment. Specifically, the application of MLT increased the content of flavonoids, flavanol, isoflavonoids, flavanones and coumarins. At the same time, a substantial decrease was observed in the presence of NaCl. Moreover, terpenes (mainly terpenoids, diterpenoids and sesquiterpenoids) were decreased by MLT treatment under non‐stress conditions. The sesquiterpenoid desoxyhemigossypol‐6‐methyl ether increased only in the presence of NaCl, mostly at the higher MLT concentrations. Modulation of phytohormones was also outlined in the samples under the higher MLT concentrations. The auxin (indol‐3‐yl) acetate decreased in all conditions considered, while the 2‐cis,4‐trans‐xanthoxin, which participates in the abscisic acid biosynthesis, and the cytokinin dyhydrozeatin, only reduced under salinity. Interestingly, the modulation of endogen melatonin was only observed at the highest concentrations (50 and 150 MLT) in a dose‐dependent manner in both salt and non‐salt conditions.

## 4 DISCUSSION

Despite the large evidence of the biostimulant effect of the exogenous application of MLT, the molecular and physiological causes supporting such an effect remain unclear. The current experimental design provides a time course determination of the photosynthetic efficiency in lettuce plants subjected to foliar‐sprayed MLT in the absence and presence of 100 mM NaCl. Being glycophytes, lettuces do not thrive under high salt stress, and 100–200 mM NaCl concentration can severely inhibit most cultivar's growth (Zhu 2001). Previous studies outlined 100 mM dosage as already positively effective in affecting leaf number, FW‐DW, chlorophyll, flavonoids, anthocyanins, and carotenoids contents, as well as the nitrogen balance in various cultivars (Kim et al. 2008; Garrido et al. 2014; Adhikari et al. 2021).

In this study, the evaluation of the biostimulant effect of exogenous MLT and induced salt stress along the 2‐week experiment was carried out by a dual approach: the combination of the PSII efficiency based on Fv/Fm, a common indicator of the maximum quantum yield of PSII chemistry (Butler 1978; Oxborough 2004), and the leaf projected area. Indeed, the reduction of Fv/Fm is related to the damage of the PSII, which results in photoinhibition, and so it is widely defined as a stress indicator in leaves (Lucini et al. 2015; Simko et al. 2016; Acosta‐Motos et al. 2017), while projected above‐ground area of lettuces revealed through the PSI system strongly correlated to leaves FW (Figure S1), as previously observed in tomato (Paul et al. 2019), thus being an indicator of the above‐ground biomass.

While the leaf area remained stable, starting from T2 and for the remaining part of the experiment, plants treated with 150 μM MLT (the highest dose tested) showed a positive modulation of Fv/Fm both in control and stressed conditions, suggesting the impact of MLT in enhancing plants' photosynthetic performance by slightly increasing the PSII electron transport (Arnao 2014). The foliar application of MLT biostimulant and the NaCl soil watering induced an imbalance at the systemic level since T3, probably due to the integration of the response to the stress and biostimulant applied. This was confirmed by the results outlined at T4, which corresponded to a stabilization of the lettuces' response to the stress. Specifically, the PSII quantum yield was unrelated to the single MLT or NaCl application but to their interaction, with NaCl +150 MLT plants reaching higher values. Despite the stressful conditions in this study, the electron flow of PSII was not inhibited, as indicated by the stable Fv/Fm values. The absence of a reduction of Fv/Fm in NaCl‐treated plants, already observed by Franzoni et al. (2022), may be due to the variety selected being strongly dependent on the genotype (Pérez‐López et al. 2013; Xu and Mou 2015) and the short duration of the stress application (14 days), thus impacting plants in later growing phases (Sorrentino et al. 2022). Meanwhile, the application of 150 μM MLT seems to positively modulate photosynthetic maximum quantum yield. This can be correlated to the increase in stomatal conductance, transpiration rates and chlorophyll *a* and *b* content mediated by the MLT application, particularly at the highest dosage (El‐Bauome et al. 2024; Kiremit et al. 2024).

The stunted growth phenotype of plants under NaCl conditions was visible at longer NaCl‐exposure times (T3 and T4), and the FW and DW of leaves and roots decreased in NaCl‐treated lettuces at T4, hence increasing leaf DM% (Table 1 and Table S2). This is due to the reduced capacity of plants to uptake water from the growing substrate under salt stress, in accordance with previous research (Xu and Mou 2015; Breś et al. 2022). In this study, MLT did not improve biomass parameters and leaf projected area in the control lettuces, according to the results reported by Shi et al. (2015). Moreover, the biostimulant did not alleviate the effects of salt treatment by significantly increasing the Fw and DW of leaves and roots, possibly because of the foliar application method and the limited impact of NaCl on below‐ground levels. Interestingly, recent works on MLT application for mitigating the salinity effect on lettuce confirmed the hierarchical effect of NaCl over MLT in affecting lettuce yield, leaf area and number of leaves while confirming the positive impact of MLT in more extended experiments (6–10 weeks) (El‐Bauome et al. 2024; Kiremit et al. 2024). Notably, 150 μM MLT was more effective than 50 and 100 μM doses, further supporting our results (El‐Bauome et al. 2024).

Considering the plant metabolic reaction, applying 50 μM and 150 μM MLT in non‐stress conditions provided a dose‐dependent response, as outlined by previous work (Zhang et al. 2015). Both the unsupervised and supervised statistical analysis suggested that the MLT treatments affected the leaf metabolome despite the hierarchically higher effect of salinity. In fact, within the salinity cluster, the stressed controls were separated from MLT treatments, thus indicating the unique impact of MLT on leaf physiology. Among the different MLT concentrations, 26 compounds were common, but the highest concentration (150 μM) was more effective in modulating the plant metabolism than 10 μM and 50 μM, with the specific regulation of 52 compounds. Therefore, melatonin, depending on the doses applied, can modulate the biosynthesis of different groups of compounds, including those involved in secondary metabolism, phytohormones, fatty acids and amino acids synthesis.

In our results, despite the general down accumulation of secondary metabolites in both MLT‐treated and control samples (Figure 6), the content of some phenylpropanoids (like flavonoids, flavanols, flavones, flavanones and coumarins) increased, suggesting their antioxidant role during stress. Aside from its direct role in the scavenging of radicals and ROS, exogenous MLT induces the activation of antioxidant enzymes by increasing the endogenous melatonin level, flavonoids such as gallic acid, iso‐quercetin and luteolin and inducing the linoleic acid and amino acid metabolism (Xie et al. 2021; Song et al. 2022; Rajkumari et al. 2023), thus increasing its efficiency as an antioxidant through both direct and indirect mechanisms (Shi et al. 2015). Similarly, the general decrease of nitrogen‐containing compounds was lower in MLT‐stressed plants than in MLT‐treated plants. These results agree with Kiremit et al. (2024), who suggested that reduction in the nitrate content induced by MLT was enhanced, besides nitrogen‐related metabolic pathways, by the altered expression in nitrate transporter genes. Previous works indicated the beneficial effect of the exogenous MLT application under different environmental stress through the modulation of primary and secondary metabolites in several species like tomato (Dou et al. 2022), soybean (Cao et al. 2020), and buckwheat (Hossain et al. 2020).

In our conditions, the exogenous melatonin also modulated the phytohormones profile with a general positive increase in 150 MLT and a decrease in lower and mid concentrations. Melatonin and indole acetic acid (IAA) are two indolamine types, with tryptophan as a precursor regulating plant growth and development (Khan et al. 2022). MLT possesses IAA‐like activities and is involved in the metabolism of different hormones like gibberellins, cytokinins and ethylene (Arnao and Hernández‐Ruiz 2018), besides mediating the biosynthesis of abscisic acid (ABA) by regulating its metabolism and reducing its signalling, thus improving plants' tolerance to adverse conditions, including NaCl (Fu et al. 2017). The enhanced intermediate of abscisic acid in 150 MLT‐treated plants might be an MLT‐induced defence mechanism to salinity, as ABA is widely recognized to reduce stomatal conductance, water consumption and transpiration to maintain low Na^+^/K^+^ and to enhance the activity of protective enzymes, ultimately keeping an intact cell membrane structure and reducing the salinity‐induced damage (Tuteja 2007). Moreover, decreased cytokinins were outlined in all MLT samples, both under salinity and non‐salinity conditions. From a genetic perspective, these phytohormones are generally negative regulators of the responses to salt stress (Yu et al. 2022). Nonetheless, different works also suggested positive effects of cytokinins in the response to NaCl (Feng et al. 2019), temperature (Wu et al. 2017) and drought (Nguyen et al. 2016).

Concerning the melatonin *x* salinity interaction outlined by AMOPLS statistics, carbohydrate derivatives (2‐*C*‐methyl‐*D*‐erythritol 4‐phosphate, deoxyxylulose 5‐phosphate and sucrose 6‐phosphate), the auxin indole‐3‐acetate, the furanocoumarin bergaptol, as well as the cysteine derivatives Se‐methyl‐selenocysteine and propenyl‐*L*‐cysteine, could be identified as main discriminants. Notably, the methylthritol 4‐phosphate pathway of isoprenoid synthesis provides compounds that act in resistance to abiotic stresses (i.e., carotenoids and isoprenoids), which defend against oxidative stress. Their production is preferred in stress conditions, including high temperatures, light, and low water supply (Perreca et al. 2020).

## 5 CONCLUSION

The positive effect of exogenous melatonin application on plant stress mitigation has been widely recognized as a promising solution for establishing crop‐resilient systems within the current worldwide climate change crisis. In this work, untargeted metabolomics and plant phenotyping were jointly employed to potentially unravel the role of MLT dosages in mitigating salinity stress using *Lactuca sativa* L. from a high throughput perspective. Our study showed that exogenous MLT affected the plant's photosynthetic performance in both controls and stress conditions from early stages, with distinctive dosage‐ and time‐dependent effects. The metabolomic approach highlighted that high MLT concentration could enhance salinity stress mitigation, following a concentration‐dependent effect. Indeed, secondary metabolism, phytohormones, fatty acids, and amino acid biosynthesis were modulated based on different exogenous MLT concentrations. High MLT dose application was found to inhibit cytokinins biosynthesis coupled with the elicitation of ABA biosynthesis, suggesting a fine‐tuning reprogramming of the phytohormonal profile to cope with the salinity‐induced responses. Overall, the present findings open a broad paradigm facing the current limitations on the study of the effects of exogenous MLT from a molecular point of view, paving the road towards implementing more efficient workflows to elucidate the biochemical basis of plant stress mitigation responses.

## AUTHOR CONTRIBUTIONS

**Elena Secomandi:** Data curation; Formal analysis; Writing ‐ original draft. **Biancamaria Senizza:** Data curation; Formal analysis; Writing ‐ original draft. **Marco Armando De Gregorio**: Data curation; Formal analysis; **Begona Miras‐Moreno**: Formal analysis. **Rosa Maria Rivero**: Supervision; review & editing. **Pascual Garcia‐Perez**: Data curation; Formal analysis; Writing ‐ review & editing. **Luigi Lucini**: Conceptualization; Data curation; Supervision; Resources; Writing ‐ review & editing.

## FUNDING INFORMATION

This research did not receive any specific grant from funding agencies in the public, commercial, or not‐for‐profit sectors.

## Supporting information


**Data S1:** Supporting Information.

**Table S3.** Comprehensive list of putatively annotated compounds identified.
Table S4. Comprehensive list of compounds resulting from supervised OPLS‐discriminant analysis.

## Data Availability

https://physiologiaplantarum.org/instructions-for-manuscrip/#Data availability statement
