# Supplementary material for: Phenotyping and metabolomics insights into the effect of melatonin in lettuce under non‐stress and salinity conditions
Source: Physiol Plant. 2025 Jan 15;177(1):e70055. doi: 10.1111/ppl.70055 (PMC11733844; doi:10.1111/ppl.70055)
Supplement: Supplementary file 1 — Data S1: Supporting Information. [file PPL-177-e70055-s002.pdf]

Phenotyping and metabolomics insights into the effect of melatonin in lettuce under non-stress and salinity conditions

Elena Secomandi[1,2§], Biancamaria Senizza[1§], Marco Armando De Gregorio[1], Begona Miras-Moreno[3], Rosa Maria Rivero[3], Pascual Garcia-Perez[1], Luigi Lucini[1*]

[1] *Department for Sustainable Food Process, Università Cattolica del Sacro Cuore, 29122 Piacenza, Italy*

[2] *Department of Sciences, Technologies and Society, University School for Advanced Studies, IUSS, Pavia, Italy*

[3] *Department of Plant Nutrition, Center of Edaphology and Applied Biology of Segura (CEBAS-CSIC), 30100 Murcia, Spain*

[§] these authors contributed equally to the work

* Corresponding author: luigi.lucini@unicatt.it

Untargeted metabolomics analysis

Chromatographic separation of root extracts and root exudates was performed in reverse phase on an Agilent Poroshell 120 PFP column (100 mm × 2.1 i.d., 1.9 µm particle size). The mobile phase consisted of ultrapure water (A) and acetonitrile (B), both acidified with 0.1% v/v of formic acid (Carlo Erba, Cornaredo, MI). A gradient elution mode was employed, starting from 6% to 94% B in 32 min. The constant flow rate was set at 0.2 mL/min, and 6 µL of the extract was injected into the system.

The QTOF mass analyzer operated in positive mode (ESI+) using the following ESI conditions: nitrogen was used as both sheath gas (12 L/min and 315°C) and drying gas (14 L/min and 250°C), nebulizer pressure was 45 psi, nozzle voltage was 350 V, and the capillary voltage was 4.0 kV. High-resolution mass spectrometry acquisition was carried out by the full scan mode within the m/z range from 100 to 1200 at a rate of 1 spectra/s with a resolution power of 30,000 full width at half maximum (FWHM) at m/z 200. The MassHunter Qualitative Analysis software (version B.06.00, Agilent Technologies) was used for chromatogram processing.

UHPLC-QTOF-MS raw data generated were processed by the MassHunter Profinder 10.0 software (Agilent Technologies) using the “find-by-formula” algorithm. The metabolomics workflow included peak finding within the retention time (RT) range of 1–32 min and m/z range of 100–1200, retention time and mass alignment, and peak annotation using a mass tolerance of 5 ppm. For feature detection, a filter for data reduction was applied, which consisted of removing features not detected in at least 75% of replications within the same sample group. The annotation of features was based on accurate mass obtained from the full scan MS data using the PlantCyc database, with a 5-ppm tolerance for mass accuracy. The annotation process considered the isotope pattern, including monoisotopic mass, isotopic spacing, and isotopic ratio. According to Metabolomics Standards Initiative (MSI), confidence Level 2 of identification (i.e., probable structures or putatively annotated compounds) was achieved through our untargeted metabolomics approach (Salek et al., 2013).

Figure S1. Correlation within Fresh Weight (g) and Leaf projected area (mm^2) acquired with PSI system at T4

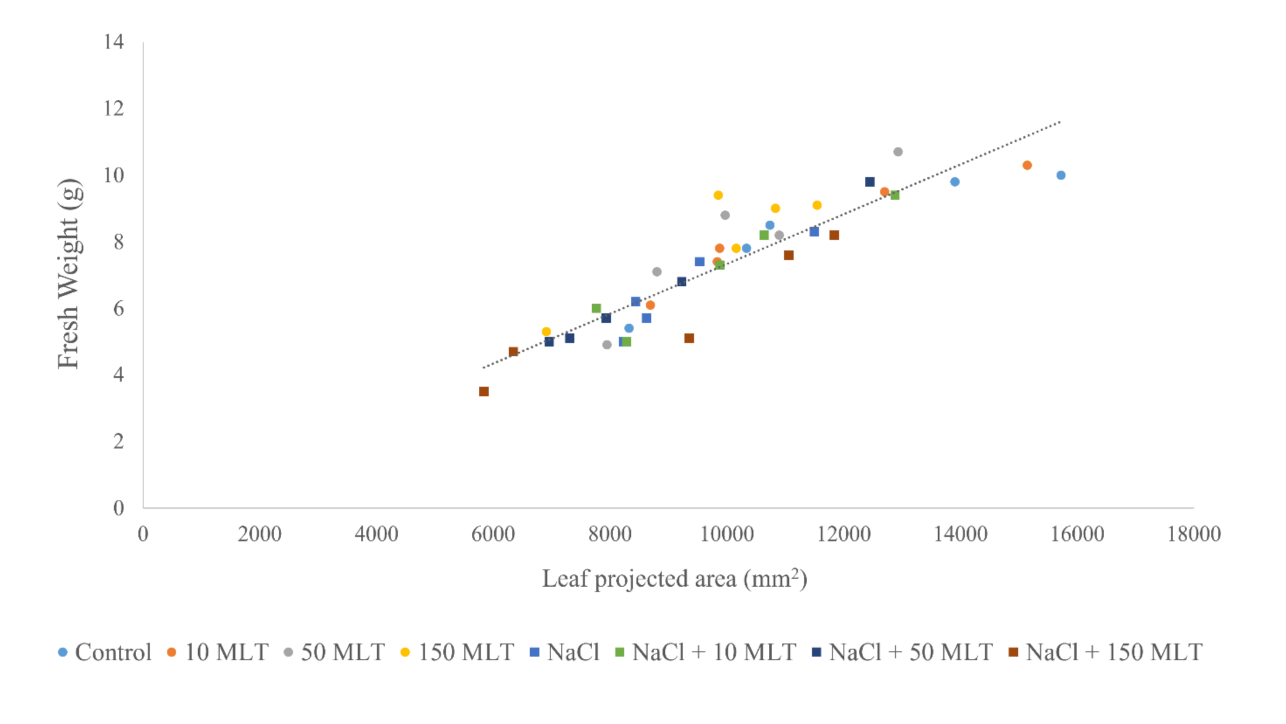

Figure S2. Effect of melatonin on the maximum quantum yield of photosystem II (Fv/Fm).

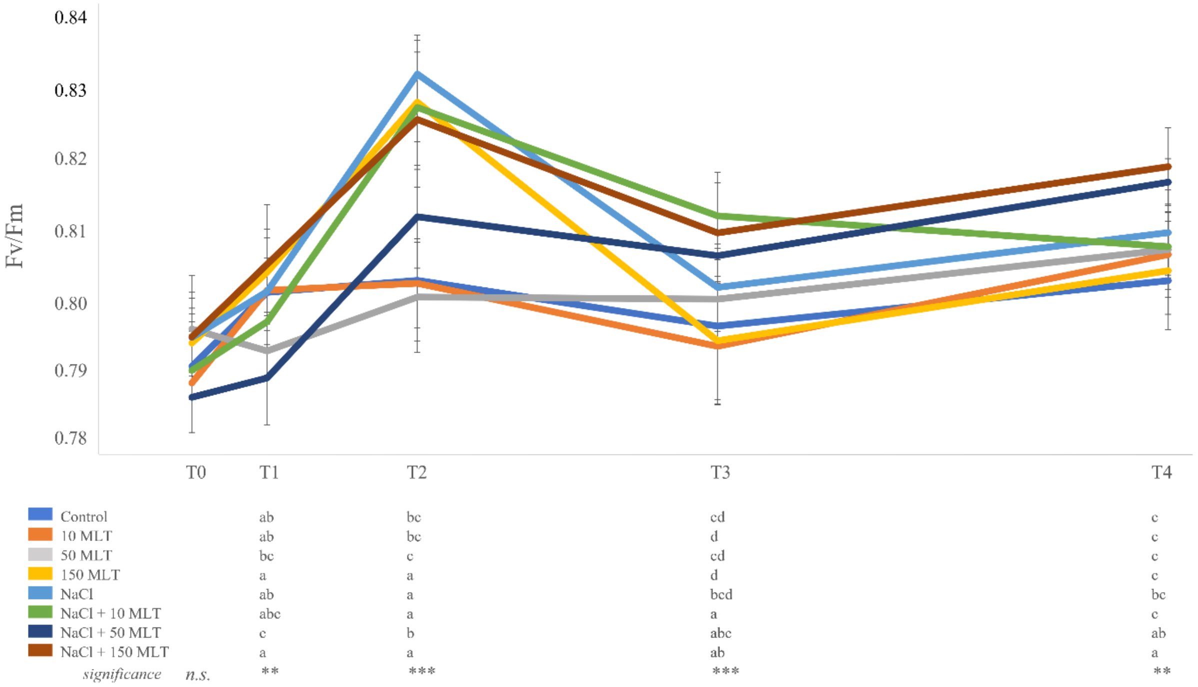

Table S**1.** Effect of melatonin concentrations (10, 50, 150 μM) and salinity (100 mM) conditions on lettuce leaves and roots (Fresh and Dry weight and Leaf Dry Matter %).

Source of variance	Leaf Fresh Weight	Leaf Dry Weight	Root Fresh Weight	Root Dry Weight	Leaf DM%
	(g Plant^{-1})	(g Plant^{-1})	(g Plant^{-1})	(g Plant^{-1})	%
**Salinity (S; mM NaCl)**					
Control NaCl	8.15 ± 1.71	0.65 ± 0.15	6.42 ± 0.84	0.36 ± 0.04	8.07 ± 0.98
100 mM NaCl	6.50 ± 1.71	0.65 ± 0.18	4.44 ± 0.81	0.26 ± 0.02	9.95 ± 0.99
	***	n.s.	***	**	***
**Melatonin (MLT; mM)**					
0	7.41 ± 1.79	0.67 ± 0.14	5.57 ± 1.24	0.35 ± 0.13	9.31 ± 1.27
10	7.70 ± 1.70	0.67 ± 0.16	5.50 ± 1.66	0.23 ± 0.08	8.84 ± 1.82
50	7.21 ± 2.10	0.64 ± 0.21	5.28 ± 0.91	0.29 ± 0.06	8.91 ± 1.33
150	6.97 ± 2.13	0.63 ± 0.17	5.36 ± 1.45	0.31 ± 0.08	9.17 ± 1.10
	n.s.	n.s.	n.s.	n.s.	n.s.

Table S2. Projected leaf area of lettuces under control and salinity (100 mM) conditions

	T0	T1	T2	T3	T4
**Salinity (S; mM NaCl)**					
Control	2385.553 ± 522.197	3264.553 ± 798.842	5488.181 ± 1063.343	8986.855 ± 1756.340	10758.665 ± 2332.668
NaCl	2333.404 ± 508.404	3083.08 ± 682.863	5095.292 ± 1110.401	7735.094 ± 1763.243	9206.003 ± 2012.626
significance	n.s.	n.s.	n.s.	*	*
**Melatonin (MLT; uM)**					
0	2645.880 ± 567.633	3537.412 ± 833.317	5782.591 ± 1084.527	8663.272 ± 1578.896	10538.244 ± 2551.272
10	2292.737 ± 345.792	3083.400 ± 570.306	5312.577 ± 734.740	8762.844 ± 1687.618	10570.724 ± 2333.662
50	2191.993 ± 640.396	2988.600 ± 869.834	5085.566 ± 1329.007ù	8028.096 ± 2244.456	9444.529 ± 2090.456
150	2307.302 ± 377.097	3085.850± 623.895	4986.213 ± 1122.533	7989.6862 ± 1969.997	9375.838 ± 2220.600
significance	n.s.	n.s.	n.s.	n.s.	n.s.
**S - MLT**					
Control - 0	2613.225 ± 776.243	3596.590 ± 1121.37	5813.26 ± 1392.221	9297.741 ± 1878.508	11809.052 ± 2966.405
Control - 10	2252.239 ± 419.532	2972.305 ± 703.330	5253.176 ± 771.935	9178.159 ± 1754.334	11252.356 ± 2634.406
Control - 50	2295.744 ± 563.080	3199.790 ± 875.659	5457.527 ± 1441.31	8911.584 ± 2280.602	10111.838 ± 1936.696
Control - 150	2381.003 ± 320.221	3289.525 ± 511.562	5428.762 ± 743.345	8559.936 ± 1563.130	9861.413 ± 1772.799
NaCl - 0	2678.534 ± 346.045	3478.234 ± 544.184	5751.922 ± 840.129	8028.802 ± 1036.284	9267.436 ± 1344.729
NaCl - 10	2333.236 ± 298.211	3194.495 ± 454.184	5371.978 ± 780.988	8347.528 ± 1707.709	9889.092 ± 2037.599
NaCl - 50	2088.242 ± 760.770	2777.417 ± 907.798	4713.606 ± 1245.322	7144.609 ± 2045.006	8777.219 ± 2229.026
NaCl - 150	2233.601 ± 451.480	2882.176 ± 714.420	4543.664 ± 1339.028	7419.435 ± 2340.014	8890.263 ± 2713.417
significance	n.s.	n.s.	n.s.	n.s.	n.s.

Table 3 and **Table 4** can be found in the attached excel file.
